# Combined large cell neuroendocrine carcinoma of the lung: case report with brief review

**DOI:** 10.1007/s12055-024-01826-2

**Published:** 2024-09-26

**Authors:** Sujata Agrawal, Paramita Paul

**Affiliations:** Department of Pathology, HBCH Varanasi and MPMMMCC, Varanasi, 221005 UP India

**Keywords:** Combined large cell neuroendocrine carcinoma, Immunotherapy, Molecular, Adenocarcinoma, Histopathology

## Abstract

Large cell neuroendocrine carcinoma (LCNEC) in the lung is an uncommon and highly aggressive type of histological variation, representing only a small percentage of all lung cancer cases. The occurrence of combined LCNEC, distinguished by the coexistence of neuroendocrine and non-neuroendocrine elements within a single tumor, is even more infrequent. A 49-year-old male presented with lytic lesion of the mandible with suspected lung metastasis. Biopsy of the mandibular lesion was reported as ameloblastic fibroma. The biopsy of the lung lesion turned out to be combined LCNEC of the lung. Further positron emission tomography (PET) evaluation showed multiple metastatic deposits in bilateral lungs, mediastinal nodes, liver, bone, adrenal, and kidney. The patient has received seven cycles of paclitaxel and carboplatin with decrease in size of nodes and lesion post 6 months. Herein, we report a case of combined LCNEC with lung adenocarcinoma which is infrequently encountered and has been a subject of research with a brief review of literature.

## Introduction

Large cell neuroendocrine carcinoma (LCNEC) is rare constituting 3% of all lung cancers and accounting for 2.1 ~ 3.5% of pulmonary surgically resected specimens [1]. Combined large cell neuroendocrine carcinoma (C-LCNEC) is characterized by the coexistence of LCNEC with either adenocarcinoma (ADC)/squamous cell carcinoma (SCC)/spindle or giant cell carcinoma, and any recognizable percentage of tumor component suffices for the definition. This combined entity constitutes more than 20–25% of resected LCNECs [2]. The C-LCNEC are usually associated with higher metastatic potential and higher stage diseases which in turn portend poor prognosis upon comparison with the pure form [3].

## Case report

A 49-year-old male with a 10-year history of tobacco intake presented with a non-healing ulcer in the right lower alveolus that had persisted for 7 months. Upon examination, an ulcero-proliferative growth measuring 5 × 2 cm involving the gingivobuccal and lingual sulcus was noted. No palpable neck nodes were present. Contrast-enhanced computed tomography (CECT) face and neck showed a lytic permeative lesion involving the alveolar process of right hemimandible from central incisor to last molar teeth with involvement of inferior alveolar canal associated with enhancing soft tissue component in right buccal space, gingivobuccal and lingual sulcus. On biopsy, it was reported as an ameloblastic fibroma.

**Fig. 1:**
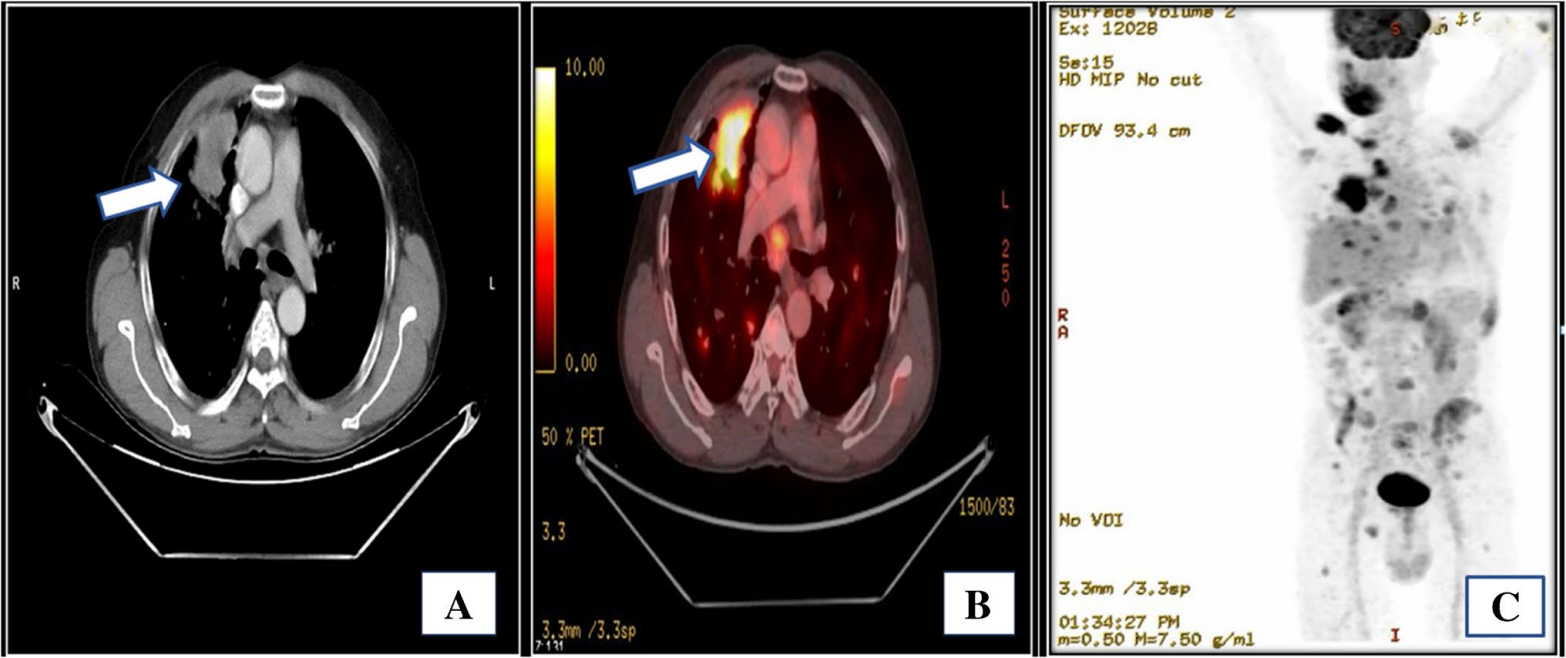
**A** and **B** F-18-FDG PET/CT (flurodeoxyglucose F18 positron emission tomography/computed tomography) scan shows FDG avid pleura-based mass in the upper lobe of right lung: ~ 68 × 48 × 64 mm, maximum standardized uptake value (SUV max) 10.9 (arrow). It abuts the right chest wall anteriorly; no obvious erosion noted. **C** Hypermetabolic metastatic lesions in bilateral lung nodule, liver, extensive sclerotic, and few lytic skeletal lesions and bilateral adrenal

High-resolution computed tomography (HRCT) of the thorax was conducted to rule out metastasis from a suspected case of oral carcinoma, revealing a 6.1 × 4.8 cm lesion in the anterior segment of the right upper lung lobe with a broad pleural interface. Additionally, multiple nodular lesions, ranging in size from approximately 3 to 10 mm, were observed in the bilateral upper right, middle, and lower lung lobes. Metastatic lymph nodes were noted in the pre-tracheal and right hilar groups. The flurodeoxyglucose positron emission tomography/computed tomography (F-18-FDG PET/CT) scan depicted a prominent pleura-associated mass in the upper lobe of the right lung, measuring approximately 68 × 48 × 64 mm (maximum standardized uptake value (SUVmax, 10.95)). Notably, the positron emission tomography (PET) scan revealed widespread metastatic involvement, encompassing the bilateral lung fields, mediastinal region, right supraclavicular nodes, liver, bilateral adrenal glands, and bones (Fig. 1).

**Fig. 2:**
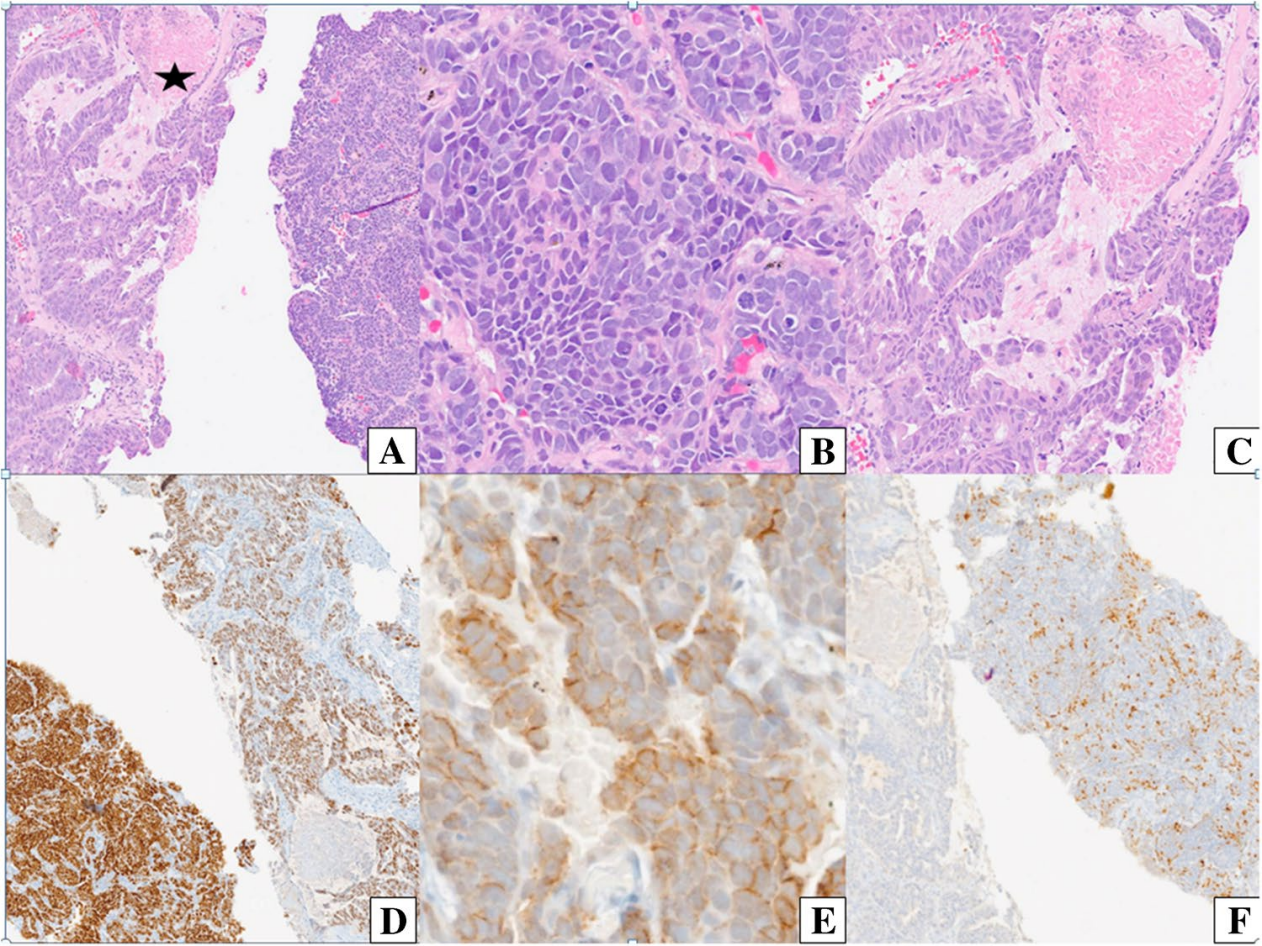
Microscopic images of combined large cell neuroendocrine carcinoma showing adenocarcinoma component (left, *) and large cell NEC (neuroendocrine carcinoma) component on the right (**A**, H&E, × 100), lobules of large-sized cells with nuclear molding (**B**, H&E, × 200), and adenocarcinoma component disposed in glandular and papillary pattern with intraglandular mucin and necrosis (**C**, H&E, × 200). Microscopic images of immunohistochemical results (**D**–**F**). Both the components are positive for thyroid transcription factor (TTF-1), NEC (neuroendocrine carcinoma) component (right) > adenocarcinoma component (left) (**D**, immunoperoxidase, × 100), (CD56) positive in neuroendocrine component (**E**, immunoperoxidase, × 40), insulinoma-associated protein 1 (INSM-1) positive in the neuroendocrine component (left) while negative in the glandular component (right) (**F**, immunoperoxidase, × 40)

A biopsy of the right upper lobe lung nodule was performed, and histopathological examination (HPE) revealed linear cores of lung parenchyma with two distinct components: glandular and LCNEC (Fig. 2A–C).

Immunohistochemistry (IHC) revealed thyroid transcription factor 1 (TTF-1) positivity in both the adenocarcinoma and LCNEC components. Additionally, the LCNEC component exhibited immunopositivity for INSM-1 and cluster of differentiation 56 (CD56), while it was negative for chromogranin and synaptophysin. The final impression was C-LCNEC with adenocarcinoma (Fig. 2D–F).

Due to the extensive metastasis of the disease, the patient commenced treatment with seven cycles of paclitaxel and carboplatin and has completed seven cycles of paclitaxel and carboplatin with a decrease in size of the mandibular as well as lung lesion. Other sites have not been evaluated yet.

## Discussion

### Classification

The World Health Organization (WHO) classifies lung neuroendocrine neoplasms into carcinoids (typical/atypical/carcinoid with high mitotic index) and carcinomas (small cell NEC, SCNEC/LCNEC, and mixed or combined carcinomas). While a 10% threshold of either component is used to diagnose combined SCNEC/LCNEC, no threshold is described for other entities. Usually, morphology itself suffices for diagnosis, but immunomarkers like insulinoma-associated protein 1 (INSM-1) (most sensitive and specific), synaptophysin, chromogranin, and CD56 (least specific) can help in the diagnosis. However, with the advent of next-generation sequencing (NGS) and several molecular updates, the NECs have undergone significant molecular reclassification. The molecular classification of SCNEC encompasses four entities: (1) small cell lung cancer (SCLC)—a characterized achaete scute family b HLH transcription factor (ASCL1) immunopositivity apart from classic neuroendocrine markers, (2) SCLC-N characterized by NEUROD1 immunopositivity and somewhat weaker expression of neuroendocrine markers, (3) SCLC-P characterized by POU2F3 (POU domain class 2 transcription factor 3) immunopositivity and absence of classic neuroendocrine markers, (4) SCLC-I/inflamed with no defined marker [4].

LCNEC represents the most heterogeneous group, encompassing three subclasses: SCLC-like LCNEC (type 1—40%), non-small cell lung cancer (NSCLC)–like LCNEC (type 2—55%), and carcinoid-like LCNEC (5%). SCLC-like LCNEC is characterized by some molecular alterations typical of conventional SCLC, such as retinoblastoma 1 (RB1) and TP53 inactivation, MYCL1 amplification, CREB binding protein (CREBBP), EP300 and KMT2A gene mutations, as well as fibroblast growth factor receptor 1 (FGFR1) amplifications; however, they differ from conventional SCLC in their transcriptomic profile (ASCL1-low/DLL3-low/Notch-high profile in SCLC-like LCNEC versus ASCL1-high/delta-like 3 (DLL3)-high/Notch-low expression profile in conventional SCLC). Conversely, NSCLC-like LCNECs share some molecular alterations with non-NE-tumors, such as cyclin-dependent kinase inhibitor 2A (CDKN2A) deletion, TTF-1 amplifications, Kirsten rat sarcoma (KRAS), Kelch-like ECH-associated protein 1 (KEAP1), and liver kinase B1 (LKB1) mutations or alterations in other rat sarcoma (RAS) pathway genes. Carcinoid-like LCNEC shares multiple endocrine neoplasia 1 (MEN1) mutations with neuroendocrine tumor (NETs) [4, 5]. The details are summarized in Table 1.

**Table 1:** Molecular classification of small cell and large cell neuroendocrine carcinoma

	Small cell lung cancer				Large cell neuroendocrine carcinoma		
Molecular subtypes	SCLC-A	SCLC-N	SCLC-P	SCLC-A	SCLC-like LCNEC (type 1)	NSCLC-like LCNEC (type 2) (55%)	Carcinoid-like LCNEC (5%)
Conventional neuroendocrine markers (INSM-1, synaptophysin, chromogranin)	Positive	Positive/negative	Negative	Negative	Positive/negative	Positive/negative	Positive/negative
Definitive transcription factor (immunohistochemistry)	ASCL1	NEUROD1	POU2F3	NA	NA	NA	NA
TTF-1	Positive	Negative	Negative	Negative		Positive/negative	Positive/negative
Mutations	TP53 RB1	TP53 RB1	TP53 RB1	TP53 RB1	TP53 RB1	CDKN2A, TTF-1 amplifications; KRAS, KEAP1, and LKB1; other RAS pathway genes	MEN1

*NA*, not available

### Diagnosis

The clinical features of LCNEC, whether pure or combined, are similar, prevalence being more in middle-aged male smokers. The signs and symptoms are nonspecific leading to a high disease and metastatic burden. Our case also had no complaining symptoms related to lung. Yang et al. attributed the clinical manifestations to the components present. They found adenocarcinomas-LCNEC to be more common in young female never-smokers, with peripheral location and driver gene mutation, while squamous-LCNEC was more common in elder male patients with a central location. However, disease-free survival (DFS) and overall survival (OS) was similar between the two C-LCNEC subtypes. The median DFS of combined SCC/LCNEC was 23.57 months and of combined adenocarcinoma/LCNEC was 24.47 months [6].

C-LCNEC is seldom reported on small biopsies due to the inadequate representation of the NSCLC component. Our case had six cores which was made in two blocks; two of the cores showed adenocarcinoma component, and one was necrotic while the others showed neuroendocrine component.

### Molecular profile

Simbolo et al. in their extensive molecular profiling of 44 cases of C-LCNEC found three clusters, CLCNEC clusters 4, 7, and 9 (CL4, CL7, CL9) by gene expression profiling. CL4 was the most heterogeneous cluster encompassing different tumor combinations with the highest chromogranin A staining and was characterized by recurrent alterations in TP53, RB1, and KRAS genes and had a hypomethylation signature. CL7 included only combined adenocarcinomas, most of which harbored mutations in KRAS and/or KEAP1 and STK11. No case displayed RB1 alterations. This cluster showed a profile quite similar to pure adenocarcinomas and to LCNEC type I. The CL9 cluster was also heterogeneous and expressed the highest values of Ki-67, TP53 and RB1 being the most frequently altered genes with absence of KRAS alteration. The epithelial-mesenchymal transition, inflammation-related signatures, and the cytotoxic T lymphocyte–associated protein 4 (CTLA4) blockade immunotherapy signature were correlated with CL9. Thus, it can be inferred that the evaluation of molecular signatures can help in deciding a targeted treatment [7]. However, NGS was not performed in the present case.

### Treatment

A second major problem with high-grade neuroendocrine lung tumors is the paucity of therapeutic options. Actionable mutations, like EGFR mutations or anaplastic lymphoma kinase (ALK) fusions, are exceedingly rare, with a frequency of < 5% in LCNEC and even lower in SCLC [7, 8]. Therefore, routine molecular workup with NGS is not mandatory for these histologies, but should be considered in the special case of a never/ex/light-smoker with < 15 pack-years, because patients with EGFR, ALK, RET, etc. can gain many months of survival with tyrosine kinase inhibitors (TKIs), according to several case reports and small retrospective series.

The treatment regimen of C-LCNEC mainly pertains to LCNEC component and includes complete surgical resection for limited-stage (I-IIIA disease). Studies have shown stage II and higher stage disease could benefit from adjuvant chemotherapy. Some studies found the benefit of adjuvant chemotherapy even in stage I disease [6]. The chemotherapy selection for advanced or metastatic C-LCNEC is a matter of debate and includes SCLC-like or NSCLC-like regimen; however, several clinical studies reported better prognosis in patients receiving SCLC regimen. More evidence is required to ascertain the same [9]. The index case received NSCLC regimen containing paclitaxel and carboplatin. Post three cycles, the mandibular lesion and lymph nodes showed a decrease in size. The evaluation of rest of the lesions is planned, but not performed yet.

Immunotherapy, which has shown promising results in NSCLC, has shown some evidence of partial response in a subset of C-LCNEC. A combination of chemotherapy, radiotherapy, and immune checkpoint inhibitors has shown partial to complete response in some case studies but more clinical data are needed to support this statement. Yan et al. in their study divided the treatment arms into two groups. Group A patients received immune check point inhibitors (ICIs) which was in turn divided into those receiving ICI in combination with chemotherapy and those receiving in combination with anti-angiogenic drugs. Group B patients did not receive ICIs. The median OS in the ICI group was 23.5 months as compared to the non-ICI group (11.23 months). ICIs consisted of pembrolizumab, camrelizumab, sintilimab, toripalimab, tislelizumab, atezolizumab, or durvalumab [10].

The NSCLC component in C-LCNEC also allows targeting the driver mutations like EGFR and ALK. Use of TKIs or ALK inhibitors can be used in this subset of patients with combined adenocarcinoma. Table 2 depicts a brief review of literature of the recent reported cases with treatment regimen [6, 11–16].

**Table 2:** Reported cases of combined large cell neuroendocrine carcinomas

Authors	Age/sex	Tumor size	Tumor combination with LCNEC	Treatment	Follow-up
Present case	49/M	NA	Adenocarcinoma	7 cycles of paclitaxel and carboplatin	Alive till date
Zirui Zhu et al., 2023 [11]	47/F	4 × 0.9 × 0.3 cm	Adenocarcinoma and SCC	Sx (right upper lobectomy), adjuvant chemo (4 cycles, docetaxel plus carboplatin)	9 months, disease progression (brain mets), DOD
Yang ZY et al., 2022 [6]		NA	Adenocarcinoma = 71 SCC = 25	NSCLC regimen (43) vs. SCLC regimen (35)	SCLC regimen had longer DFS and OS
Chloe A. Lim et al., 2022 [12]	61/M	1.5 × 2.3 cm	Adenocarcinoma	Right lower lobe lobectomy, adjuvant chemo (4 cycles, cisplatin and etoposide), alectinib	Disease progression (liver and bone mets)
Jian Xu et al., 2021 [13]	54/M	NA	Adenocarcinoma and SCLC	Sx (wedge resection), concurrent radiotherapy and chemotherapy, immunotherapy (durvalumab)	12-month, complete remission
Xiong X et al., 2021 [14]	33/M	10 × 9 × 8 cm	Fetal adenocarcinoma	Right upper lobectomy and lymph node dissection	NA
Oda R, et al., 2020 [15]	60/M	4.5 cm	SCC	Right lower lobectomy with mediastinal lymph node dissection	8 months DFS
Matsumoto T et al., 2015 [16]	79/M	NA	MALT lymphoma	Concurrent radiotherapy and chemotherapy	Disease progression (liver and bone mets)

*LCNEC*, large cell neuroendocrine carcinoma; *Adeno*, adenocarcinoma; *SCC*, squamous cell carcinoma; *Sx*, surgery; *NSCLC*, non-small cell lung carcinoma; *SCLC*, small cell lung carcinoma; *DFS*, disease-free survival; *OS*, overall survival; *NA*, not available; *MALT*, mucosa-associated lymphoid tissue; *DOD*, died of disease

## Conclusion

C-LCNEC with adenocarcinoma is a rare and highly aggressive cancer. The treatment regimen of C-LCNEC is not yet clear despite the development of targeted therapy and NGS. Awareness of this subset is crucial for future research and devising a treatment approach.
